# Hand Biometric Recognition Based on Fused Hand Geometry and Vascular Patterns

**DOI:** 10.3390/s130302895

**Published:** 2013-02-28

**Authors:** GiTae Park, Soowon Kim

**Affiliations:** ASIC Design Lab, Department of Electrical Engineering, University of Korea, Seoul 136-701, Korea; E-Mail: pgtbk@asic.korea.ac.kr

**Keywords:** multimodal biometric, hand biometric, hand geometry, vascular-pattern recognition

## Abstract

A hand biometric authentication method based on measurements of the user's hand geometry and vascular pattern is proposed. To acquire the hand geometry, the thickness of the side view of the hand, the K-curvature with a hand-shaped chain code, the lengths and angles of the finger valleys, and the lengths and profiles of the fingers were used, and for the vascular pattern, the direction-based vascular-pattern extraction method was used, and thus, a new multimodal biometric approach is proposed. The proposed multimodal biometric system uses only one image to extract the feature points. This system can be configured for low-cost devices. Our multimodal biometric-approach hand-geometry (the side view of the hand and the back of hand) and vascular-pattern recognition method performs at the score level. The results of our study showed that the equal error rate of the proposed system was 0.06%.

## 1. Introduction

The rapidly growing biometric recognition industry [1] requires that its systems deliver high-security in applications such as computer systems and limited-access control areas. Biometrics is the term used in the computer sciences to refer to the field of mathematical analysis regarding unique human features. Hand biometrics is a relatively new type of biometric system. Various biometric features of the hand can be extracted; these are: hand geometry [2–6], finger knuckle [7], vascular pattern of the fingers [8,9], and the vascular pattern of the hand [10–17]. The features of unimodal biometrics have many limitations, such as variation in an individual biometric feature. In order to overcome using unimodal biometrics, combinations of multimodal biometrics [18–21] are being widely developed.

Table 1 presents the relation between the features for identification, the population involved together with results obtained, in terms of performance (FAR: False Acceptance Rate, FRR: False Rejection Rate, EER: Equal Error Rate). Best results in Table 1 are achieved by [21] and our work with multi-modal biometrics. Our work presents a new approach to achieve improved performance (EER = 0.06).

Our study proposes a multimodal biometric approach integrating hand geometry and vascular patterns. Our proposed multimodal biometric system can be constructed as a low-cost device because our system uses only one image to extract the feature points. We perform multimodal biometrics by score-level fusion with z-score normalization, which results in improved recognition performance compared to that of unimodal biometrics consisting of each hand geometry (e.g., the side view of the hand and the back of hand) and vascular pattern.

The rest of this paper is organized as follows: in Section 2, we discuss the hand biometric recognition system and we talk about the proposed hand biometric recognition technique. In Section 3, we discuss the experimental results. We conclude in Section 4.

## 2. Experimental Section

### 2.1. Hand Biometric Recognition System

In this section, we discuss the hand biometric recognition system. A proposed user-authentication system using the side and back view of the hand is investigated. The implemented system is detailed in Section 2.1.1. Details of the acquisition device are provided in 2.1.2. The image segmentation and preprocessing are illustrated in Section 2.1.3.

#### 2.1.1. Overview

**Figure 1.**
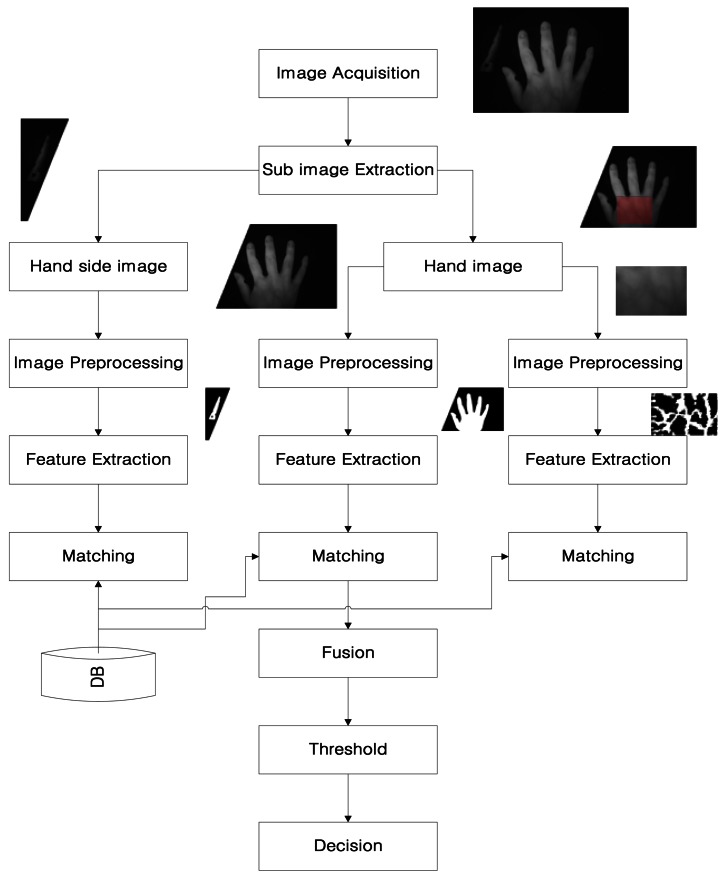
Block diagram of the implemented system.

**Figure 2.**
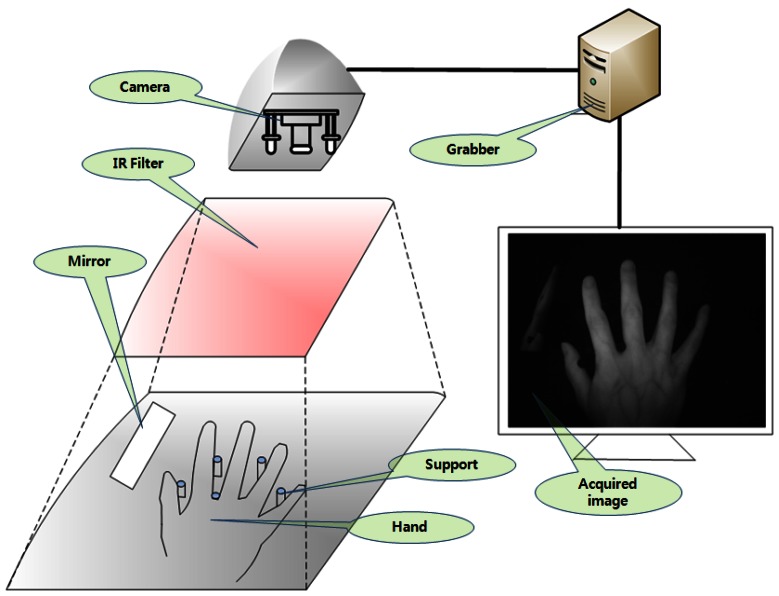
Acquisition of a sample image of the back of a hand.

The block diagram of the implemented system is shown in Figure 1. First, a hand image is obtained from an acquisition device consisting of camera equipped with an infrared (IR) Light-Emitting Diode (LED), IR filter, mirror, and support for the hand, as shown in Figure 2. The camera video signal (analog output) is converted into an image (digital signal) through a grabber board. To extract hand geometric features and hand vascular patterns from the acquired image, we perform hand segmentation by a predetermined area between the side view of the hand and the back of hand. The next step is to search the region of interest (ROI) for the vascular pattern. The vascular pattern is separated from the back of the hand. The extracted sub-image is composed of the three (side view of the hand, the back of hand, and the vascular pattern). Then, feature points are extracted after preprocessing. The matching is calculated using feature points between the data base (DB) and those of the sub-image. The matching score of the side view of the hand, the back of hand, and the vascular pattern is calculated using the Euclidean distance, the distance measure for polygonal curves, and template matching. Finally, we combine these three scores using score-level fusion based on z-score normalization.

#### 2.1.2. Acquisition Device

An acquisition system has been developed for the collection of the side- and back-of-the-hand data and the vascular-pattern-of-the-hand data to acquire a single image. An acquisition device as shown in Figure 2 is constructed. That device is illuminated by a fixed light source located above the hand. The resolution of the acquired image is 640 × 480 pixels.

The acquisition of a sample image is shown in Figure 2. For the work on hand-based biometric identification, an IR LED (840∼850 nm) was used. An input image is captured in an IR environment to acquire the hand vascular pattern. To prevent movement of the hand a fixed support device was used. In order to take the side-of-the-hand image, a mirror was installed. A camera with a Charge-Coupled Device (CCD) sensor (1/3 type B/W) changes light signals into electrical signals. The light signals contain visible light (400–700 nm) and the near-infrared region. An IR filter (850 nm) removes the unwanted light wavelengths and is used to extract vein patterns.

#### 2.1.3. Image Segmentation and Preprocessing

**Figure 3.**
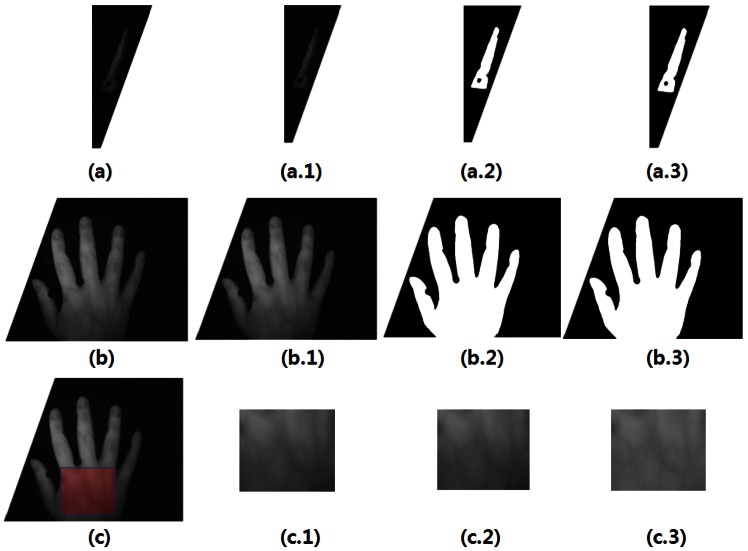
Preprocessing for hand recognition. (**a**) the side view of the hand, (**a.1**) Gaussian filter, (**a.2**) threshold, (**a.3**) median filter; (**b**) the back-of-the-hand view, (**b.1**) Gaussian filter, (**b.2**) threshold, (**b.3**) median filter; (**c**) the region of interest (ROI) of vascular, (**c.1**) vascular image, (**c.2**) Gaussian filter, and (**c.3**) high-pass filter.

First, for hand recognition, the hand image is captured, and then preprocessing is performed. Preprocessing is conducted in two steps: (1) the gray image is transformed into a black and white one where the background is eliminated. The preprocessing for the side view of the hand is shown in Figure 3(a). The preprocessing for the back-of-the-hand data is shown in Figure 3(b). And, (2), the noise is removed in order to begin the vascular-pattern extraction (VPE) algorithm, as shown in Figure 3(c). Figure 3(a.1),(b.1),(c.2) show the Gaussian filter for noise removal. Figure 3(a.2),(b.2) show the threshold. Figure 3(a.3),(b.3) show the median filter for noise reduction of the threshold image. Figure 3(c.3) shows the high-pass filter for emphasizing the vascular patterns.

**Figure 4.**
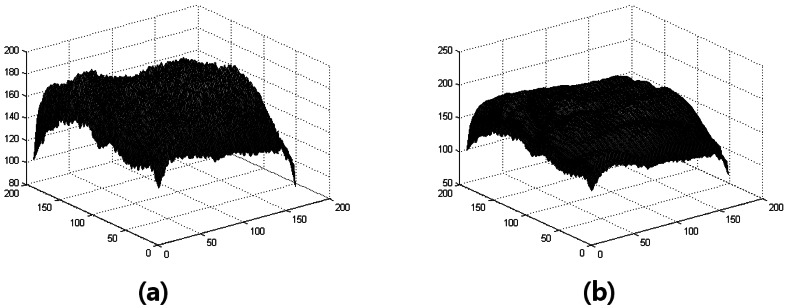
Gaussian filter. (**a**) Vascular image; (**b**) Image after Gaussian filter.

The Gaussian smoothing can be performed using standard convolution methods. The image has *M* rows and *N* columns, and the kernel has *m* rows and *n* columns. We use a suitable integer-valued convolution kernel that approximates a Gaussian with a *σ* of 1. Gaussian filtering is shown in Figure 4.

The 2D Gaussian is expressed as:

(1) $$G(x,y)=\frac{1}{2\pi \sigma^{2}}e^{-\frac{x^{2}+y^{2}}{2\sigma^{2}}}$$

**Figure 5.**
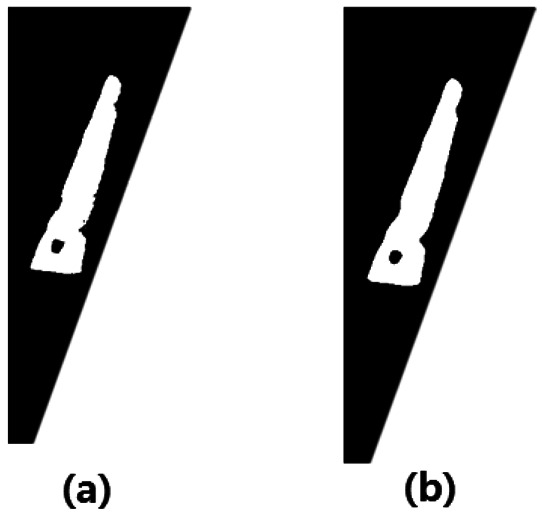
Median filter. (**a**) Threshold image; (**b**) Image after Median filter.

The median filter is to compare these results to a threshold value. The input data is thereby converted to a binary value (0,1). The images of Vascular, Median filter are shown in Figure 5.

The median filter is expressed as:

(2) $$\begin{array}{l} z(x_{c},y_{c})=1,\text{if}\sum_{x=1}^{M}\sum_{y=1}^{N}z(x,y)\geq K\\ z(x_{c},y_{c})=0,\text{if}\sum_{x=1}^{M}\sum_{y=1}^{N}z(x,y)<K \end{array}$$

The next step after preprocessing is the extraction of the feature points. The extraction of the feature-points process includes the thickness of the side view of the hand, the K-curvature [22,23], and the vascular pattern.

### 2.2. Proposed Hand Biometric Recognition Technique

This section addresses the algorithm used for hand biometric recognition. We detail the extraction of feature and verifier. The side view of the hand is detailed in Section 2.2.1. The back-of-the-hand view is provided in Section 2.2.2. The VPE are illustrated in Section 2.2.3.

#### 2.2.1. The Side View of the Hand

**Figure 6.**
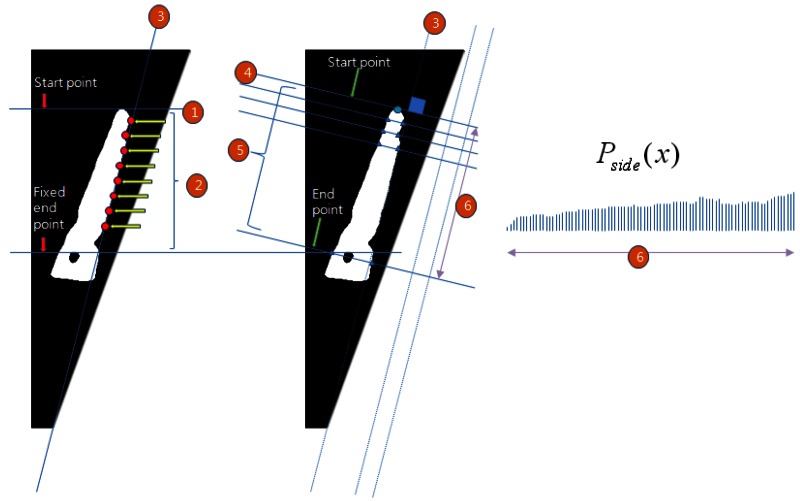
Thickness search of the side view of the hand and the profile of the thickness.

To establish the thickness of the side view of the hand, the heights of the middle finger, the index finger, and the palm are collected and calculated in the following order: (1) find a line at the base of the palm; (2) next, find the starting point perpendicular to the palm base line; (3) then, calculate the thickness of the side view the hand from the starting point to the end point. The location of the endpoint is predetermined by the acquisition device. The profile of thickness is *P_side_*(*x*), as shown in Figure 6.

#### 2.2.2. The Back of the Hand

The curvature can define a curve intwo-dimensional space. The curvature of the discrete data in a digital image using a suitable approximation is obtained. The concept of K-curvature is such that a continuous curvature is represented by a discrete function.

**Figure 7.**
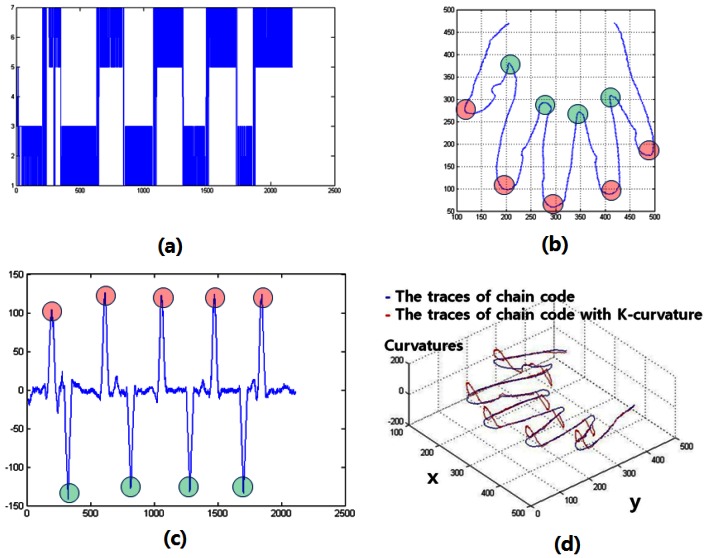
(**a**) the chain code; (**b**) traces of the chain code; (**c**) K-curvature, and (**d**) trace of the chain code with K-curvature.

In this study, the K-curvature uses the curvature of the boundaries of the hands and the background as feature vectors. The K-curvature is calculated in the following order: (1) The chain code representation of the hand surface pattern is obtained. The traces of chain code are represented by blue in Figure 7(b),(d); (2) Then, the K-curvature is calculated using data from the trace of chain code.

The K-curvature is expressed as:

(3) $$\kappa (p_{i},k)=\frac{1}{k}\sum_{j=-k}^{-1}f_{i-j}-\sum_{j=0}^{k-1}f_{i-j}$$

The curvature at a point *p_i_* is taken as the difference between the mean angular direction of *K* vectors on the leading curve segment of *p_i_* and that *K* of vectors on the trailing curve segment of *P_i_f_i_* is the *i th* component of the chain code. K-curvature begins at the beginning of a thumb.

The traces of the K-curvature are represented by red in Figure 7(d) (*k* = 30). The “maximum peak” means the end of the finger [marked in red in Figure 7(b),(c)]. The “minimum peak” values mean the contact point between the fingers [marked in blue in Figure 7(b),(c)].

**Figure 8.**
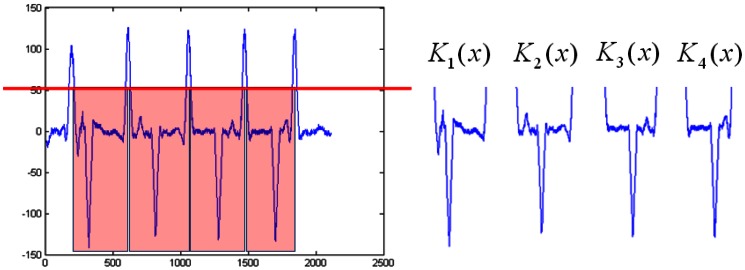
The feature extraction for K-curvature.

The first feature of the hand geometry is the divided K-curvatures. The original K-curvature is split into components that can be characterized. These components consist of *K_1_*(*x*) for the valley between the thumb and index finger; *K_2_*(*x*) for the valley between the index and middle fingers; *K_3_*(*x*) for the valley between the ring and index fingers; and *K_4_*(*x*) for the valley between the ring and little fingers. The features of the end of each finger were removed by a K-curvature above 50. Figure 8 shows the feature extraction for the K-curvature.

**Figure 9.**
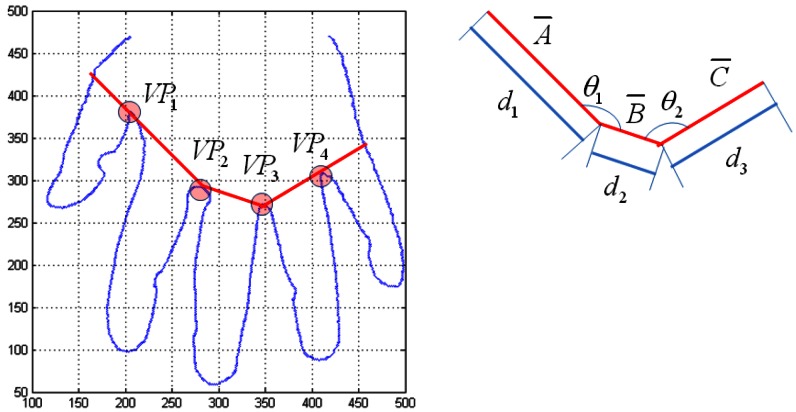
The feature extraction for lengths and angles of finger valleys.

The second feature of the hand geometry is the length and the angle of the finger valley that is calculated by the K-curvature. Valley points consist of *VP_1_*, *VP_2_*, *VP_3_*, and *VP_4_*. The *d_1_* is length of *a̅* that is connected from *VP_2_* to the outer edge of the hand through *VP_1_*. The *d_2_* is the length of *b̅* that is connected from *VP_2_* to *VP_3_*. The *d_3_* is the length of *c̅* that is connected from *VP_3_* to the outer edge of the hand through *VP_4_*. The *θ*_1_ is the angle between *VP_1_* and *VP_3_* on the basis of *VP_2_*. The *θ*_2_ is the angle between *VP_2_* and *VP_4_* on the basis of *VP_3_*. Figure 9 shows the feature extraction for the lengths and angles of the finger valleys.

**Figure 10.**
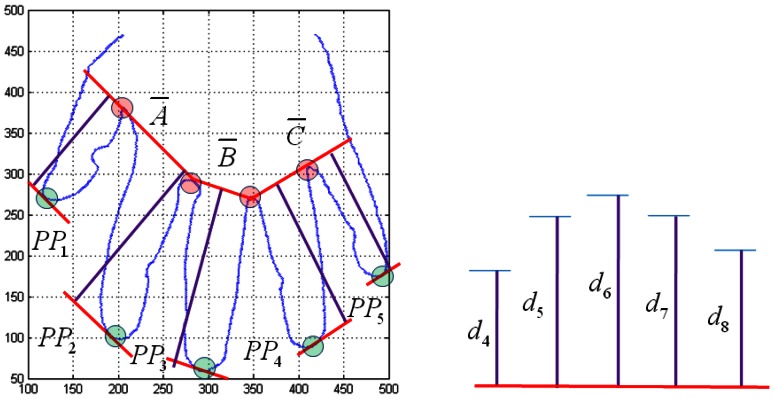
The feature extraction for the lengths of fingers.

The third feature of the hand geometry is the length of the fingers. The peak points for K-curvature consist of *PP_1_*, *PP_2_*, *PP_3_*, and *PP_4_*. The lengths are *d_1_* from *PP_1_* to *a̅* ; *d_5_* from *PP_1_* to *a̅* ; *d_6_* from *PP_2_* to *a̅* ; *d_7_* from *PP_1_* to *a̅* ; and *d_8_* from *PP_1_* to *a̅* The *d_4_* is the length of the line from *PP_1_* to *a̅*. The *d_5_* is the length of the line from *PP_2_* to *a̅*. The *d_6_* is the length of the line from *PP_3_* to *b̅*. The *d_7_* is the length of the line from *PP_4_* to *c̅*. The *d_8_* is the length of the line from *PP_5_* to *c̅*. Figure 10 shows the feature extraction for the lengths of the fingers.

**Figure 11.**
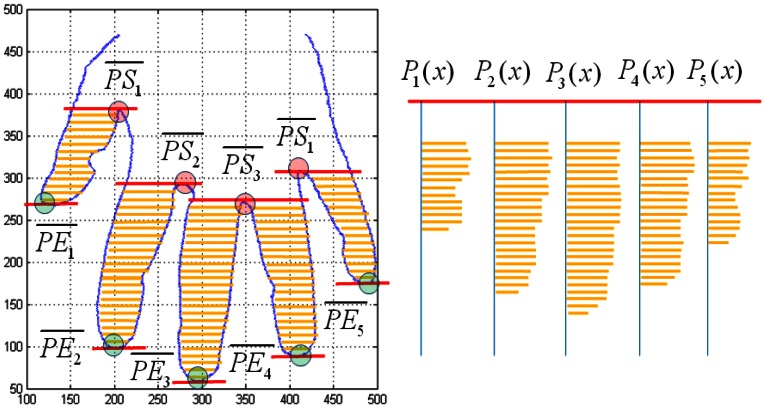
The feature extraction for the profile of fingers.

The fourth feature of hand geometry is the profile of the fingers. The starting points of the profile are the y-axis coordinates at the valley points. The end points of the profile are the y-axis coordinates at peak points. The starting point of the baseline consists of $\bar{PS_{1}}$, $\bar{PS_{2}}$, $\bar{PS_{3}}$, and $\bar{PS_{4}}$. The end point of the baseline consists of $\bar{PP_{1}}$, $\bar{PP_{2}}$, $\bar{PP_{3}}$, $\bar{PP_{4}}$, and $\bar{PP_{5}}$. The profile of the fingers consists of *P*_1_(*x*), *P*_2_(*x*), *P*_3_(*x*), *P*_4_(*x*), and *P*_5_(*x*). Figure 11 shows the feature extraction for the profile of fingers.

#### 2.2.3. VPE

**Figure 12.**
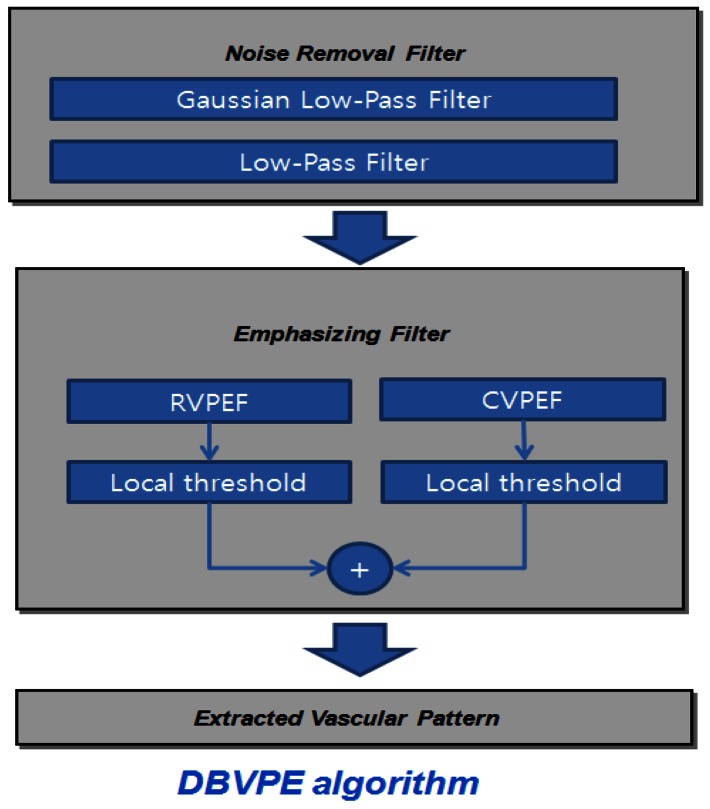
The VPE algorithm processing.

The VPE algorithm is implemented by using the direction-based vascular-pattern extraction (DBVPE) method [11]. The DBVPE uses a noise-removal filter that consists of a Gaussian low-pass filter and a smoothing low-pass filter. The DBVPE uses an emphasizing filter that combines the output of the row VPE filter (RVPEF) and the column vascular-pattern extraction filter (CVPEF). The VPE processing is illustrated in Figure 12.

**Figure 13.**
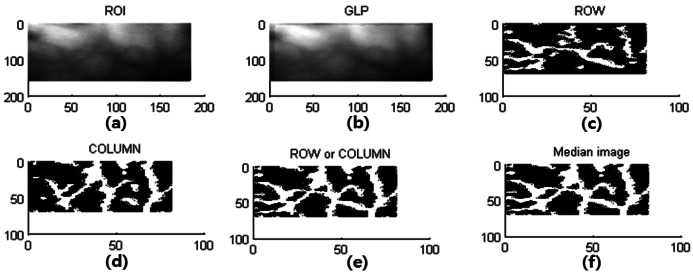
Curvatures estimate. (**a**) the ROI for preprocessing of vascular pattern; (**b**) Gaussian filter; (**c**) row vascular-pattern extraction filter; (**d**) column vascular-pattern extraction filter; (**e**) row or column vascular-pattern; and (**f**) median filter.

The VPE algorithm uses a noise-removal filter and an emphasizing filter. The VPE algorithm is shown in Figure 13(c–f). The VPE is calculated in the following order: (1) first, for the noise removal filter, a Gaussian low-pass filter was used, as shown in Figure 13(b); (2) then, the emphasizing filter combines the output of the RVPEF in Figure 13(c); (3) next, the CVPEF in Figure 11(d); (4) next, RVPEF and CVPEF are added together in Figure 13(e); (5) finally, The median filter for noise reduction is shown in Figure 13(f).

The emphasizing filter is expressed as:

(4) $$z(x_{c},y_{c})=\sum_{x=1}^{M}\sum_{y=1}^{N}z(x,y)w(x-x_{c},y-y_{c})$$

with *w*(*x,y*) being equal to

(5) $$w(x,y)=S_{\left(x,y\right)}2^{-k_{\left(x,y\right)}}$$

where *z*(*c*), *_M_*, *_N_*, and *w*(*i*) are the center pixels of the filter mask, the abscissa vector size of the mask, the ordinate vector size of the mask, and the filter coefficient of the emphasizing filter, respectively. *S*(*x,y*) is one of {−1,1}, and *k*(*x,y*) is any integer number. We used RVPEF with an 11 × 17 kernel with horizontal characteristics, and CVPEF with an 11 × 17 kernel with vertical characteristics.

#### 2.2.4. The Features of Hand Recognition

The feature of hand recognition is illustrated in Table 2. These features are the length, angle, profile, and vascular pattern, and they are used as data for verification.

#### 2.2.5. Matching

In order to compare the different features of hand recognition, three kinds of verifier algorithms are used. The first algorithm is Euclidean distance. To establish the angle and length, the Euclidean distance algorithm was used. It performs its measurements with the following equation:

(6) $$D=\sqrt{\sum_{i=1}^{L}{\left(X_{i}-Y_{i}\right)}^{2}}$$

with *L* being the dimension of the feature vector; *X_i_* is the *ith* component of the source feature vector; *X_i_* = {*θ_t_*_1_,*θ_t_*_2_,*d_t_*_1_,*d_t_*_2_,*d_t_*_3_,*d_t_*_4_,*d_t_*_5_,*d_t_*_6_,*d_t_*_7_,*d_t_*_8_} ; and *Y_i_* is the *ith* component of the target feature vector; *Y_i_* = {*θ_s_*_1_,*θ_s_*_2_,*d_s_*_1_,*d_s_*_2_,*d_s_*_3_,*d_s_*_4_,*d_s_*_5_,*d_s_*_6_,*d_s_*_7_,*d_s_*_8_}.

The second algorithm is the distance measured for the polygonal curves. For the K-curvature and profile, the distance-measure algorithm was used. An approach to a distance measurement for polygonal curves is to make a comparison between the original curves and the target curves with the objective of minimizing some property under specific constraints on the possible mappings; this algorithm performs its measurements with the following equation:

(7) $$\delta_{i}([P_{i}],[Q_{i}])=\underset{h,s\in \mathbb{R},s>0}{\min}\int_{-\infty }^{\infty }\left|sk_{P_{i}}(st-h)-k_{Q_{i}}(t)\right|dt$$

where *P_i_* is the *ith* component of the source feature vector:

$$P_{i}=\{P_{s\_\text{side}}(x),P_{s1}(x),P_{s2}(x),P_{s3}(x),P_{s4}(x),P_{s5}(x),K_{s1}(x),K_{s2}(x),K_{s3}(x),K_{s4}(x)\}$$

*Q_i_* is the *ith* component of the target feature vector:

$$Q_{i}=\{P_{t\_\text{side}}(x),P_{t1}(x),P_{t2}(x),P_{t3}(x),P_{t4}(x),P_{t5}(x),K_{t1}(x),K_{t2}(x),K_{t3}(x),K_{t4}(x)\}$$

*h* is shifted along the x-axis to minimize the integral; *s* is scaled uniformly by any positive values;

For the K-curvature and profile, the number of scores is 10.

The third algorithm is a matching algorithm. The matching algorithm is used for the vascular pattern, and it obtains the maximum matching value between the source patterns and target patterns. The patterns consist of the vascular pattern and the background pattern. The matching of patterns is calculated by giving a weight of 1/4. The third algorithm performs its measurements with the following equation:

(8) $$C(s,t)=\frac{\sum_{x}\sum_{y}f(x,y)w(x-s,y-t)}{\sum_{x}\sum_{y}f(x,y)}\times \frac{1}{4}+\frac{\sum_{x}\sum_{y}\bar{f}(x,y)\bar{w}(x-s,y-t)}{\sum_{x}\sum_{y}f(x,y)}\times \frac{1}{4}+\frac{\sum_{x}\sum_{y}f(x,y)w(x-s,y-t)}{\sum_{x}\sum_{y}w(x,y)}\times \frac{1}{4}+\frac{\sum_{x}\sum_{y}\bar{f}(x,y)\bar{w}(x-s,y-t)}{\sum_{x}\sum_{y}\bar{w}(x,y)}\times \frac{1}{4}$$

where *f*(*x,y*) is the vascular value of the source pattern; *w*(*x,y*) is the vascular value of the target pattern; *f̅*(*x*,*y*) is the background value of the source pattern; *w̅*(*x*,*y*) is the value background of the target pattern; *s* is the matching point of the x-axis; *t* is the matching point of the y-axis.

Three kinds of verifier algorithms compute 12 matching scores. The 12 matching scores are illustrated in Table 3.

At the verifier state, the source templates are compared with the target template. A source or target template is represented by 21 feature vectors: one profile of thickness, four K-curvatures, two angles, eight lengths, five profiles of fingers, and one vascular pattern. Angle and length are grouped into a single matching score. The verifier between the source templates and the target templates consists of computing 12 matching scores between them.

For hand geometry recognition, we used a weighted sum between the Euclidean distance and the distance measurement for polygonal curves. The weights *W*_1_ and *W*_2_ are varied over the range [0,1] in steps of 0.01, such that the constraint *W*_1_ + *W*_2_ = 1 is satisfied. The best weights for the Euclidean distance and the distance measurements are 0.37 and 0.63. Measurements are performed using the following equation:

(9) $$V_{\text{Hand}\_\text{geometric}}=0.37\times D+0.63\times (\delta_{1}+\delta_{1}+\delta_{1}\ldots +\delta_{10})$$

The VPE recognition performs its measurements with the following equation:

(10) $$V_{\text{VPE}}=C$$

The false acceptance rate (FAR) is the error rate of accepting the wrong person; the false reject rate (FRR) is the error rate of rejecting own; the genuine acceptance rate (GAR) is 1 − FRR; and the equal error rate (EER) is the error rate when FRR is equal to the FAR.

#### 2.2.6. Matching

Multimodal biometric uses various levels of fusion: matching-score level, decision level, and the feature-extraction level. In this paper, we used integration at the matching-score level. The matching-score level comprises two approaches: the classification approach and the combination approach. Because the combination approach performs better than some classification approaches [24], we select the combination approach that combines the individual matching scores to generate a single scalar score.

The matching-score level needs normalization to transform the score into a common domain before combining it. In this paper, normalization uses a z-score [25]. The z-score is calculated using the arithmetic mean and standard deviation of the given data.

The normalized scores are expressed as:

(11) $$s_{k}^{\prime }=\frac{s_{k}-\mu }{\sigma }$$

where *μ* is the arithmetic mean, and *σ* is the standard deviation.

**Figure 14.**
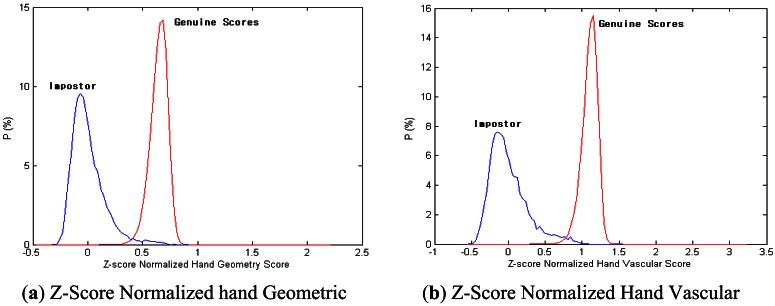
Distribution of genuine and impostor scores after z-score Normalization (**a**) hand geometry; (**b**) hand vascular pattern.

The distributions of the matching scores of the two modalities after z-score normalization are shown in Figure 14.

Once normalized, the normalized-scores obtained from hand geometric and vascular pattern are combined using a simple weighted-summation operation. The weighted-summation method is given by:

(12) $$F=pS_{h}+(1-p)S_{v},0\leq p\leq 1$$

where *S_h_* and *S_v_* are the normalized-scores of the hand geometric and vascular pattern, respectively, and *F* is the fused-score. *p* is the weight of the normalized-scores obtained from hand geometric authentication, while (1 − *p*) is the weight of the normalized-scores obtained from vascular pattern. In the decision, the fusion score is compared with the decision threshold *T*. When the fusion score *F* is greater than *T*, the person is recognized; otherwise the person is not.

## 3. Results

The experimental database contains a total of 1,300 images (side-view-of-the-hand, back-of-the-hand and vascular-pattern-of-the-hand images) for 100 subjects, *i.e.*, 13 images per individual. We use three images each individual (a total of 300 images) for training. To test the proposed recognition method, we use 10 images per hand from 100 people. For training and test purposes, each of these biometric data sets is partitioned into with 3 × 100 and with 10 × 100 samples. The users' ages ranged from 20 to 50. Approximately 73% were men, and 27% were women.

**Figure 15.**
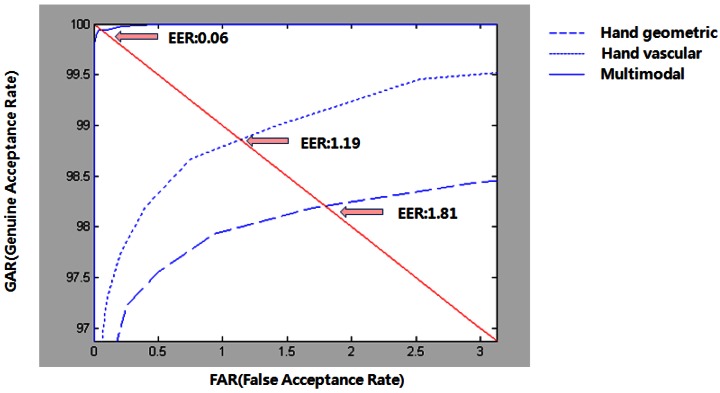
ROC curves of unimodal and multimodal biometrics.

In our experiments, we use summed score of all the scores from each unimodal matching as a final matching score. As the EER of unimodal biometrics, hand geometry, and VPE acquired 1.81%, and 1.19%. Our proposed approach is based on a score-level fusion with the unimodal biometrics approach. The score level was normalized as a z-score. The fusion of hand geometry and the VPE obtains the best EER of 0.06%. Figure 15 shows the ROC curves for unimodal and multimodal biometrics.

We measured the speed of the proposed algorithm on a desktop computer with Intel Pentium (R) Dual CPU 2.00 GHz processor, with 2.00 GB of RAM The computational complexity of processing is summarized in Table 4.

## 4. Conclusions

In this article, we have proposed a new multimodal biometric verification method based on the fusion of the hand geometry and the vascular pattern from a single hand image. The proposed hand recognition method was based on K-curvature, thickness of the side view of the hand, and VPE. The accuracy of the proposed multimodal biometrics method is better than that obtained using unimodal biometrics.

## Figures and Tables

**Table 1.** Comparative biometric example (dorsum hand geometry, dorsum hand vascular pattern and multi-model biometrics).

**Year [Ref.]**	**Type**	**Features**	**Population size**	**Performance [%]**
1999 [2]	H	Contour coordinates	53	FAR = 1, FRR = 6
1999 [3]	H	Length, width, thickness and deviation	20	EER = 5
2006 [4]	H	Width and Curvature	73	EER = 3.6
2009 [5]	H	Fusion SVDD	86	EER = 1.5
1995 [14]	V	Sequential correlation	20	FAR = 0, FRR = 7.9
2004 [15]	V	Feature points of the vein patterns	32	EER = 2.3
2004 [16]	V	FFT based phase correlation	25	FAR = 0.73, FRR = 4
2005 [17]	V	Distance between feature points	48	FAR = 0, FRR = 0.9
2009 [18]	VK	Vascular structures and knuckle shape	100	EER = 1.14
2003 [19]	PH	Palm-print and Hand Geometry	100	FAR = 0, FRR = 1.41
2003 [20]	PH	Palm-print and Hand Geometry	50	FAR = 0.1818, FRR = 1
2010 [21]	VF	Vascular and geometry of finger	102	EER = 0.075
Our work	VH	Vascular and geometry of hand	100	EER = 0.06

H: Hand geometry, V: Vascular, K: Knuckle shape, P: Palm-print, F: Finger geometry.

**Table 2.** The features of hand recognition.

	**Features**
The side view of the hand	**profile of thickness:***P_side_*(*x*)
The back-of-the-hand view	**K-curvature:***K*_1_(*x*), *K*_2_(*x*), *K*_3_(*x*), *K*_4_(*x*)
	**angle:***θ*_1_, *θ*_2_
	**LENGTH:** *d*_1_, *d*_2_, *d*_3_, *d*_4_, *d*_5_, *d*_6_, *d*_7_, *d*_8_
	profile of fingers: *P*_1_(*x*), *P*_2_(*x*), *P*_3_(*x*), …. *P*_4_(*x*), *P*_5_(*x*),
VPE	**vascular pattern**

**Table 3.** The matching score.

	**Matching score**
Euclidean distance	*D*
Distance measurement for polygonal curves	*δ*_1_, *δ*_2_, *δ*_3_, *δ_4_*, *δ*_5_, *δ*_6_, *δ*_7_, *δ*_8_, *δ*_9_, *δ*_10_
matching	*C*

**Table 4.** The computational timing for processing.

**Processing**	**Time (msec)**
Image Preprocessing	112
Hand geometric Processing	11
VPE Processing	16
